# RATTUS (Rat Thoracic Ultrasound): diagnosis of pneumothorax in pet rats

**DOI:** 10.3389/fvets.2024.1394291

**Published:** 2024-09-13

**Authors:** A. Piskovská, K. Kraszewska, K. Hauptman, J. Chloupek, P. Linhart, V. Jekl

**Affiliations:** ^1^Jekl & Hauptman Veterinary Clinic, Brno, Czechia; ^2^Department of Pharmacology and Pharmacy, Faculty of Veterinary Medicine, VETUNI, Brno, Czechia; ^3^Vetcardia Veterinary Clinic, Warsaw, Poland; ^4^Department of Animal Protection and Welfare and Veterinary Public Health, Faculty of Veterinary Hygiene and Ecology, VETUNI, Brno, Czechia

**Keywords:** rat, RATTUS, pneumothorax, thoracic disease, respiratory disorders, dyspnoea, ultrasonography

## Abstract

**Introduction:**

Rat thoracic ultrasound (RATTUS) is a non-invasive, easy-to-perform method for the evaluation of the pleural space and lungs in pet rats. The aim of the article is to present species-specific differences in the sonographic diagnosis of pneumothorax (PTX) in pet rats.

**Methods:**

In total, 158 client-owned pet rats were examined during the period from July 2023 to January 2024. PTX was diagnosed in 20 of the examined rats (13.25%, the age of the animals ranged from 2 months to 32 months (19.08 ± 6.93 months; mean ± SD) and their body weight ranged from 97 g to 885 g (461.27 ± 138.97 g; mean ± SD). Radiographic confirmation of PTX was performed in all these 20 rats, in the control group radiography was used to confirm that PTX was not present.

**Results:**

The lung point and the barcode sign was found in 7/20 animals with sensitivity of 33.3% (95% CI, 0.16–0.59) and specificity of 100% (95% CI, 0.97–1.0). The abnormal curtain sign was found in 19/20 of animals with the sensitivity of 95% (95% CI, 0.73–0.99.7) and the specificity of 89% (95% CI, 0.82–0.93). The abnormalities in the substernal access were in 17/20 of animals with the sensitivity of 85% (95% CI, 0.61–0.96) and the specificity of 71% (95% CI, 0.62–0.78).

**Discussion:**

In conclusion, RATTUS is a non-invasive method for the diagnosis of PTX in rats. Lung point and barcode sign are specific but not easily diagnosed signs. The curtain sign in RATTUS is not specific for PTX, as there are e.g. geriatric rats (rats older than 1,5 years) in which the abnormal curtain sign is visible without the presence of PTX. The presence of moderate to severe PTX can be assessed by the substernal approach based on the presence of cardiac displacement toward the collapsed lung lobe, and on evaluation of the lung inflation symmetry. This sign is not specific for PTX but in conjunction with other ultrasonic signs described makes the RATTUS a feasible tool for PTX diagnosis in rats.

## Introduction

A pneumothorax (PTX) is defined by the presence of air in the pleural space, resulting in a loss of negative subatmospheric subpleural pressure and partial or complete lung collapse (1). There are two main types of spontaneous PTX: primary, when there is no apparent lung disease, secondary, when there is underlying pleural or pulmonary disease. Secondary spontaneous PTX can be caused by many different diseases such as pneumocystosis, mycobacteriosis, necrotising pneumonia, interstitial lung disease, or fibrosis. The main clinical features described in human medicine are dyspnoea, chest pain, cyanosis, hypoxaemia, and hypercapnia (2–4). Identification of PTX at the bedside is of upmost importance for appropriate and time-sensitive management of critically ill patients (5).

POCUS findings that can indicate the presence of a PTX include disappearance of lung sliding (glide sign), absence of vertical artifacts (B-lines), absence of a lung pulse, visualization of the lung point, an abnormal curtain sign and a barcode sign on M-mode (6). The presence of lung sliding along the pulmonary–pleural interface indicates sliding of the parietal and visceral pleura against each other that rules out PTX (7). The lung point is defined as the site within the thorax where the visceral pleura of the lungs recontacts the parietal pleura of the thoracic wall (8). It can sometimes be difficult to find the lung point as the air in the thorax moves with the position of the animal and in some cases the refusal chamber covers the entire surface of the lung, creating a thin air-coat, that makes it impossible to find the lung point. It cannot be observed in patients with PTX with complete collapse of the lung and its detection may be difficult in patients with diffuse alteration of lung sliding of aetiology other than PTX [(9), e.g., absceding pneumonia in rats].

**Figure 1:**
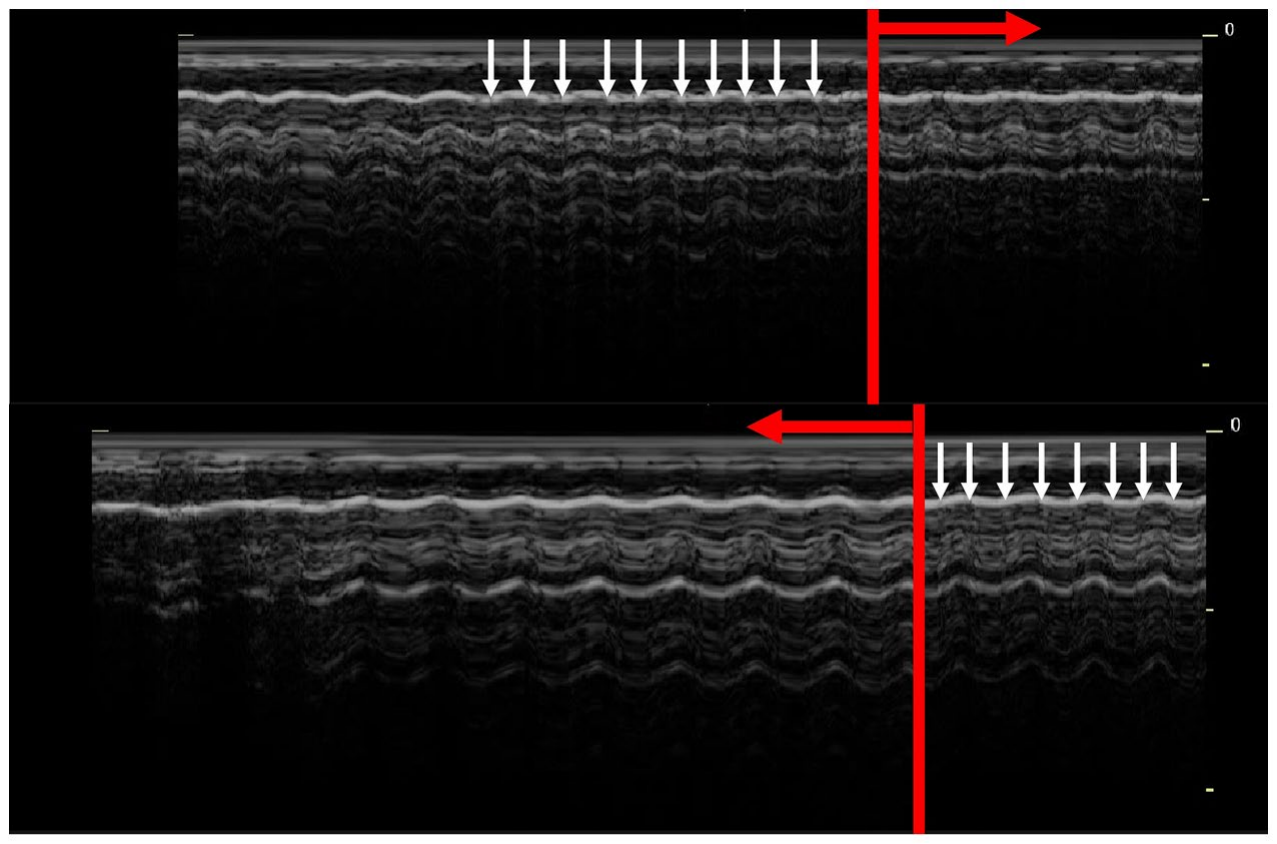
Lung point in M-mode. The barcode sign in dyspnoeic rats is normally almost indistinguishable from a physiological seashore sign. The presence of T-lines (white arrows) excludes a pneumothorax. In areas where no T-lines are present (red arrow), the lung point is confirmed.

The lung pulse is defined as the rhythmic movement of the visceral pleura in opposition to the parietal in synchrony with the cardiac rhythm. The presence of a lung pulse must be assessed because it rules out the PTX. The lung pulse presence in M-mode is called T-lines. T-lines are vertical lines going from the pleural line to the bottom of the image (10). The M-mode can be used to detect the motion of the lung sliding. When the absence of lung sliding is identified, the M-mode is performed with the cursor situated over the pleural line. Normally, the seashore sign is visualized (sandy appearance of the lungs). The absence of lung sliding is visualized as a stratosphere or barcode sign (no “sandy” appearance, only 1 pattern of straight horizontal lines, 8). Keeping distressed dogs and cats calm and still, which is required with M-mode can be very challenging and this may be a reason why M-mode is not utilized as often as B-mode for detection of PTX in companion animals (11) (Figure 1).

B-lines are vertical hyperechoic artifact that move in synchrony with the lung sliding. The presence of B-lines rules out PTX in that region, however the absence of B-lines does not confirm the diagnosis of PTX (11).

Another sonographic sign that indicates PTX is the so-called abnormal or asynchronous curtain sign. The aerated lungs and abdominal organs (usually liver or stomach) should move synchronously with inspiration and expiration (like a curtain moving through the window – lung expansion during inspiration pushing caudally the liver/stomach). An asynchronous or abnormal curtain sign can be described as the abdominal organs’ movement in the opposite direction, or asynchronous to the breathing cycle. If the abdominal content is surrounded on both sides by the aerated lung, we speak of a double curtain sign. These signs help to prove the diagnosis of PTX (11).

Recently, a methodology for thoracic ultrasound in rats was published and named RATTUS—which stands for Rat Thoracic Ultrasound (12). The aim of the article is to present species-specific differences in the sonographic diagnosis of PTX in pet rats, compared to published studies of dogs, cats (8), and horses (13). We hypothesized that similar signs with additional signs as a substernal access and evaluation of the lung inflation symmetry and positioning of the heart could be used to confirm or exclude the diagnosis of PTX in rats.

## Materials and methods

### Case selection and inclusion criteria

In total, 162 client-owned pet rats (99 female, 63 male) were examined during prospective study with the owner consent during the period from July 2023 to January 2024 in a private veterinary clinic Brno, Czech Republic. The age of the animals ranged from 2 months to 32 months (18.98 ± 6.88 months; mean ± SD) and their body weight ranged from 97 g to 885 g (461.59 ± 138.05 g; mean ± SD). All pet rats underwent general physical examination prior the diagnostic workup. Inclusion criteria were: either clinical signs or abnormal thoracic auscultation indicative for respiratory disease (dyspnea, tachypnea, crackles, wheezes) and 4-view thoracic radiographs. Exclusion criteria include absent or incomplete radiographic and ultrasonographic thoracic examination (uncooperative or unstable patients).

### Equipment

A linear multifrequency probe (8–14 MHz, SonoScape, S22, China, with MI 0.5–0.7, TIS 0.1) was used to assess the thoracic cavity. The examination was performed on conscious (non-sedated) animals. In all rats, it was not necessary to shave the hair if a generous amount of ultrasound gel was used. The examiner carefully restrained the rat with one hand while the other hand held the probe.

A special “lung preset” was used to identify artifacts on the lung surface, pleura and lung sliding. In this preset, the harmonics were turned off, the lowest frequency of the probe (8 MHz) was used, the persistence time was set to zero, the focus position was set to the height of the pleural line and increased time gain compensation (TGC) was used in the distal (far) field of the screen. These settings produce a “coarser” image (14). The study was performed in 2D and in M-mode. For all animals, the video loops and images were routinely saved for possible further analysis.

Radiography was performed in all animals included in the study with exposition 66 kV and 0.12 ms (Gierth HFX90V, Japan, 25 mA) in 4 standard position (left and right latero-lateral, ventro-dorsal and dorso-ventral), exposed to thorax.

### The RATTUS scanning technique

The thoracic ultrasound examination protocol was performed as was described by Piskovska et al. (12) in pet rats.

To avoid missing a smaller areas of PTX, it was essential to examine the entire thoracic cavity. To be sure that the whole thoracic cavity was precisely examined, scanning must be performed bordered by exact anatomical structures.

**Figure 2:**
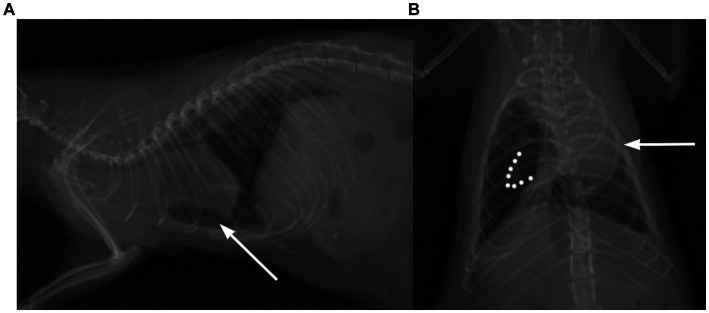
Thoracic radiogram of the rat with visible signs of pneumothorax. **(A)** The heart is lifted dorsally from the sternum in the lateral view (white arrow) and **(B)** displaced to the side of the collapsed lung lobe in the dorsoventral view (white arrow). Dotted lines demarcate collapsed lung.

The substernal access was used to assess the symmetry of the lung inflation as a crucial part of PTX diagnostics. The probe was placed perpendicular to the sternum with the sternum in the centre of the probe surface. The probe was then moved from the cranial to the caudal border with special attention to the area of the heart. Attention must be given to precise placement of the probe to the sternum because oblique placement of the probe might create a picture of false positive heart shift (Figures 1, 2).

### Observation indicators

Based on literature reports and our experiences, the following major observation indicators were used (1) the lung point, (2) the barcode sign (absence of T-lines in M-mode), (3) abnormal curtain sign, and (4) abnormalities in substernal access. The lung point was visualized as the interruption in lung sliding. While this interruption was observed, the M-mode examination was applied at this site to confirm by barcode the presence of an air in the pleural cavity. The barcode sign is not easily visualized in rats due to their high respiratory rate, thus evaluation was targeted to the absence of T-lines. The curtain sign was evaluated from the dorsal to the ventral border. In the substernal access the lung inflation symmetry was evaluated (pleural lines in plane) and the heart positioning was observed [physiologically 2/3 of the heart is on the left side, 1/3 on the right, (12)]. When the heart was deviated to the side, region of the atelectatic lung lobe was assessed in this side.

### Data collections

Standard protocol was written for each patient, including body weight, age, reason for checkup, clinical findings (all of the organ systems included), diagnostic modalities with detailed description of the performed RATTUS examination (findings in each line – axillar and scapular, left and right, mediastinum and substernal access), and therapeutical plan. Data from standard protocol and therapeutical plan were collected but were not used for this study. For each line the loop was recorded, findings supportive for PTX were recorded. The length of the loop was 20 s. Thoracic cavity evaluation was performed by single operator (AP with 2 years of lung ultrasound examination trained by KK and human medicine internal doctor specialized in lung ultrasound). Loops were recorded for each diagnosed sign and blinded, evaluated by second operator (KK–experienced clinician, with over 10 years performing echocardiographic examination and 5 years of experience in lung ultrasound with advanced training from human medicine internal doctor specialized in lung ultrasound). Radiographic images were obtained in four planes (latero-lateral left and right, dorso-ventral, ventro-dorsal) and evaluated by three observers (VJ, AP, KK) to confirm or exclude the presence of signs indicating PTX. Gas opacity in the pleural space, with no lung markings (with or without mediastinal shift, lung atelectasis and lung retraction) was considered indicative for pneumothorax (Figure 2).

### Statistical methods

Specificity and sensitivity of the lung point, the barcode sign, the abnormal curtain sign and abnormalities in substernal access for individuals with pneumothorax and without pneumothorax and area under the curve (AUC) were calculated using the Receiver Operating Characteristic curves (ROC) with use of online tool for ROC analysis (Eng, J. ROC analysis: web-based calculator for ROC curves, available from: http://www.jrocfit.org). Confidential intervals were calculated using VassarStats (online). Statistical significance was set at level *p* = 0.05.

## Results

One hundred fifty-one rats (93 female, 58 male) met the inclusion criteria. The age of the animals ranged from 2 months to 32 months (19.08 ± 6.93 months; 89 mean ± SD) and their body weight ranged from 97 g to 885 g (461.27 ± 138.97 g; mean ± SD).

**Figure 3:**
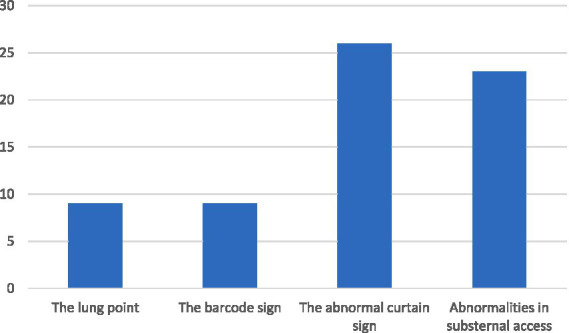
Pneumothorax diagnostics of each rat was settled by observing signs marked in the graph.

All included rats were examined by RATTUS and the diagnosis was confirmed by 4-views (latero-lateral left and right, dorso-ventral, ventro-dorsal) radiographic images. PTX was diagnosed in 20 of the examined rats (13.25%, 13 female, 7 male, their age ranged from 5 to 32 months, 19.45 ± 6.45, bodyweight ranged from 230 g to 885 g, 446.75 ± 169.19). Specificity and sensitivity for each RATTUS PTX sign was evaluated. The ultrasonographic signs detected are summarized in Table 1 and Figure 3.

**Table 1:** RATTUS signs of PTX.

RAT	The lung point	The barcode sign	The abnormal curtain sign	Abnormalities in substernal access
1*	+	+	+	+
2	+	+	+	+
3	−	−	+	+
4	−	−	+	+
5	−	−	−	+
6*	+	+	+	+
7*	+	+	+	+
8	−	−	+	+
9*	−	−	+	+
10*	−	−	+	+
11	−	−	+	+
12*	−	−	+	+
13*	−	−	+	+
14*	+	+	+	+
15	−	−	+	+
16*	+	+	+	−
17	−	−	+	−
18	−	−	+	+
19*	+	+	+	−
20*	−	−	+	+
TOTAL	7	7	19	17
Sensitivity (%)	35 (95% CI, 0.16–0.59)	35 (95% CI, 0.16–0.59)	95 (95% CI, 0.73–1.0)	85 (95% CI, 0.61–0.96)
Specificity (%)	100 (95% CI, 0.97–1.0)	100 (95% CI, 0.97–1.0)	89 (95% CI, 0.82–0.93)	71 (95% CI, 0.62–0.78)
Total number of non pneumothoracic rats where the sign was observed	0	0	15	38

The PTX diagnosis of each patient was determined based on the signs highlighted in the table. Geriatric patients are marked with an asterisk.

### The lung point

The lung point (Supplementary Video S1) was found in 35% (7/20 animals). The lung point had an overall AUC of 0.675. The 95% CI (confidential interval) of the overall effects of diagnostic accuracy estimated a sensitivity of approximately to 35% (95% CI, 0.16–0.59) and specificity of 100% (95% CI, 0.97–1.0).

The barcode sign was performed only in animals with proven lung point thus the results are identical to the lung point.

### The abnormal curtain sign

The abnormal curtain sign (Supplementary Video S2) was found in 95% of animals (, 19/20 animals). The abnormal curtain sign had an overall AUC of 0.918. The sensitivity for the presence of an abnormal curtain sign was from 95% (95% CI, 0.73–0.99.7) and the specificity was 89% (95% CI, 0.82–0.93).

### Abnormalities in the substernal access

The abnormalities in the substernal access (Supplementary Video S3) were in 85% of animals (17/20 animals). The abnormalities in the substernal access had an overall AUC of 0.78. The sensitivity for the presence of abnormalities in the substernal access was 85% (95% CI, 0.61–0.96) and the specificity was 71% (95% CI, 0.62–0.78).

## Discussion

Only a few cases of PTX have been published in exotic companion mammal medicine: a case report of lung emphysema and pneumopericardium in a rabbit (15), iatrogenic tension PTX in a rabbit as an anaesthetic complication (16), and spontaneous PTX in four pet rabbits (17) and one PTX rabbit case report (18). In a study by Guillerit et al. (16), the diagnosis was confirmed by computed tomography (CT) and thoracic radiography. The diagnosis of PTX in rats has mainly been published in laboratory animals (19, 20). In pet rats, PTX was described in a study by Fouriez-Lablée et al. (21), where the radiographs were compared with the post-mortem examination.

In two pet rabbits with PTX histopathological examination revealed chronic multifocal granulomatous pneumonia and a histiocytic sarcoma (16). Pulmonary emphysema characterised by loss of alveolar septa was described in a study by Cooper et al. (22) in many (30/36) older rabbits without respiratory signs on histopathology, suggesting that pulmonary emphysema may be a predisposing factor for spontaneous PTX (21). In the author’s experience, secondary spontaneous PTX is the most common type observed in rats due to their susceptibility to respiratory tract infections (23–26).

Cole et al. (22) published a study in which they compared CT with thoracic ultrasound in the diagnosis of PTX in dogs and cats. The sign used to confirm PTX on ultrasound was the absence of lung sliding (glide sign). PTX was confirmed in 1/3 of cases diagnosed by CT only when the VET BLUE^®^ access was used suggesting that Vet BLUE^®^ has poor sensitivity for PTX detection, as previously reported for the TFAST protocol (6, 27). TFAST protocol on the contrary has potential to rapidly diagnosed PTX as the overall sensitivity and specificity was 78.1 and 93.4% using lung sliding sign and step sign with proposed algorithm based on more than only one sign as our study propose. Vidal et al. (28) performed a retrospective study where the PTX diagnosis was evaluate by lung ultrasound compared to TXR. In this study the diagnosis of PTX was made in more cases by TXR however they used only lung point as a criteria to make sonographic diagnosis. This finding is in agreement with our results where the lung point as a single parameter would not diagnosed precisely the PTX in rats. In the human literature, meta-analysis showed that thoracic Point-of-Care Ultrasound (POCUS) has a sensitivity of 87% and a specificity of 99%, compared to thoracic radiographs (29) but the data varies among studies and used sonographic protocols (30–33). Thoracic POCUS is especially suitable for diagnosis of PTX in critically ill patients and neonates (7, 29, 34, 35). Similarly to human studies, the data varies among studies, used sonographic protocols and criteria used to assess PTX (8, 27, 36).

In RATTUS, the determination of lung sliding may be impaired due to the movement of the rats, so that the combination of more ultrasonic signs is recommended for PTX diagnosis. Detection of abnormal curtain sign improves the sensitivity of thoracic ultrasound for the diagnosis of PTX (37). Hwang et al. (36) conducted a study in which the results of lung ultrasonography based on the presence of the reverse sliding sign (abnormal curtain sign in our study) and M-mode evaluation were more accurate than the results of radiographic examination for detecting mild experimentally induced PTX in beagles. In that study the reverse sliding sign was found to be a better indicator of mild PTX with 100% specificity. In our study, the absence of the lung sliding had also lower sensitivity as in a study presented by Hwang et al. (36) and better specificity and sensitivity were for the abnormal curtain sign. But we also found this abnormal curtain sign in some geriatric rats (rats more than 18 months of age) (38) in which the curtain sign was abnormal without other signs of PTX. This could be explained by the presence of fibrotic lung tissue or chronic parenchymal lung disease (39), which produces an abnormal artifact due to changes in the diaphragm (40, 41) (Supplementary Video S4). In our study, the curtain sign had a specificity 88.5%, as the abnormalities in the curtain sign were observed in cases of non-pneumothoracic rats. In the author’s experience, it is sometimes challenging to correctly assess the curtain sign in RATTUS, especially in dyspnoeic and tachypnoeic rats (Supplementary Video S5). Slowing the loop helped to decide whether the curtain sign was normal or not. In our study, the lung point was not as good sign of PTX compared to the study by Hwang et al. (36), but this can be explained by the small size of the animals and the amount of air in the pleural space. The sensitivity is higher when the amount of air is larger, but the lung point cannot be found when the pleural air covers the entire surface of the lung, and forms a thin coating, that makes it impossible to find the lung point.

In RATTUS, more signs (lack of lung sliding, abnormal curtain sign, cardiac displacement, and asymmetry of lung inflation using substernal access) are described to detect PTX, making the examination results much more accurate. So called “lung preset” is used to evaluate RATTUS as we are focusing on artifacts and we need to use preset with harmonics turn off to be able to see all of the artifacts created by pathologically changed lung tissue. In all cases radiography was performed to confirm the sonographically diagnosed PTX. In control group, the radiographic control was used to exclude PTX in those animals. In contrast to medicine in dogs and cats, rats have to be anaesthetized in order to obtain a meaningful radiographic image and in dyspnoeic animal, the anaesthesia might be dangerous. According to this anaesthesia risk and results of this study, authors recommend to preferably perform lung ultrasound as a diagnostic test in pneumothoracic rats. Interpretation of radiographic findings might be sometimes challenging in an anaesthetised rats due to anaesthesia induced gravitational atelectasis which cause artifacts that might be falsely interpret as pathological finding (42–44).

In a substernal access, cardiac displacement may be caused by other disorders than is PTX, such as the presence of a mediastinal mass or a larger lung abscess, but these findings are usually easily recognised (Supplementary Video S6).

The size of the animals and the respiratory rate made the examination challenging for an inexperienced surgeon. The examination is well tolerated by the rats, but cannot be carried out for too long in dyspnoeic animals due to the animals’ respiratory distress and the ALARA principle have to be followed (44). The ultrasound machines must be equipped with a good quality linear multifrequency probe and offer the possibility to slow down the recorded loops and see the curtain sign in slow motion.

Limitations of this study are that the radiography was used as a gold standard confirmation method instead of the CT. This method was chosen due to its availability and due to the fact, that the high respiration rate in rats makes the CT examination challenging to evaluate. Another limitation is that mild PTX might be present in dorsal regions, especially while the animal is in sternal recumbency, and these subtle changes might be missed by substernal access but as the whole thoracic cavity was examined using more accesses and as the thoracic cavity of rats is rather small, it is very unlikely to miss those changes.

In conclusion, RATTUS is a non-invasive method for the diagnosis of PTX in rats. Lung point and barcode sign are specific signs. The curtain sign in RATTUS is not specific for PTX, as there are, e.g., geriatric rats in which the abnormal curtain sign is visible without the presence of PTX. The presence of PTX can be assessed by the substernal approach based on the presence of cardiac displacement, and on evaluation of the lung inflation symmetry. This sign is not specific for PTX but in conjunction with other ultrasonic signs described makes the RATTUS a feasible tool for PTX diagnosis in rats.

## Data Availability

The original contributions presented in the study are included in the article/Supplementary material, further inquiries can be directed to the corresponding author.

## Supplementary material

SUPPLEMENTARY VIDEO S1 Lung point: the lung point is a point where the visceral and parietal pleura touch and the lung sliding becomes visible after the interruption. Lung sliding is a “shimmer” of the pleural line and in this video the “shimmer” is interrupted (arrow) with the edges forming the lung point (https://1drv.ms/v/s!AglSmr1cGU9agSJk2I2QO6POySoT).

SUPPLEMENTARY VIDEO S2 Abnormal (asynchronous) curtain sign: the movement of the lung and abdominal contents is in opposite directions and is slowed down in the loop for easier diagnosis confirmation (https://1drv.ms/v/s!AglSmr1cGU9agSNXomomFj51XU2p).

SUPPLEMENTARY VIDEO S3 Substernal approach for the diagnosis of pneumothorax (https://1drv.ms/v/s!AglSmr1cGU9agSaoE8Wgn4Tq1iC_).

SUPPLEMENTARY VIDEO S4 Geriatric rat (rat older than 1,5 year). In geriatric rats the curtain sign can sometimes be abnormal without other signs of pneumothorax. This could be explained by the geriatric changes in the lungs, such as fibrotic lung tissue or parenchymal lung disease (https://1drv.ms/v/s!AglSmr1cGU9agS-EQry6Qu-1hAUs).

SUPPLEMENTARY VIDEO S5 Tachypnoeic rat. In tachypnoeic rats it is sometimes challenging to assess the curtain sign. Slowing down the loop helps to clearly asses the normality of the curtain sign (https://1drv.ms/v/s!AglSmr1cGU9agSR_7M94R_mAPknO).

SUPPLEMENTARY VIDEO S6 Non-pneumothoracic cardiac displacement (heart shift/cardiac displacement). The heart may be displaced from the substernal view due to a lung abscess or neoplasm and these signs are usually easy to recognize (https://1drv.ms/v/s!AglSmr1cGU9agSf3OKvvn4wzWFm3).
